# 14-type HPV mRNA test in triage of HPV DNA-positive postmenopausal women with normal cytology

**DOI:** 10.1186/s12885-020-07498-6

**Published:** 2020-10-23

**Authors:** Katrin Christine Asciutto, Christer Borgfeldt, Ola Forslund

**Affiliations:** 1 Department of Obstetrics and Gynecology, Skåne University Hospital, Lund University, SE-22185 Lund, Sweden; 2 Department of Laboratory Medicine Region Skåne, Lund University, Lund, Sweden

**Keywords:** HR-HPV mRNA testing, Cervical cancer screening, Postmenopausal women

## Abstract

**Background:**

During 2013 and 2016 the region of Skåne, Sweden started to analyse human papillomavirus (HPV) and cytology in postmenopausal women 60–65 years of age. Our aim was to evaluate high-risk (HR) HPV mRNA testing for the triage of HPV DNA-positive postmenopausal women with normal cytology.

**Methods:**

A total of 271 women, 60–65 years of age, underwent liquid-based cytology (LBC) and HPV testing by using the HR-HPV DNA MGP-PCR-Luminex assay. HR-HPV DNA-positive women with normal cytology underwent complimentary HPV mRNA testing (Aptima, Hologic Inc.). Over a period of 49 months (SD 11.0) the women received regular follow-ups at intervals of 12–18 months. Women with abnormal cytology and/or a positive HR-HPV DNA and/or mRNA result at two subsequent visits were scheduled for colposcopy and clinical examination.

**Results:**

Over the surveillance period, 3.6% (10/271) of the HR-HPV DNA-positive women developed histologically confirmed high-grade squamous intraepithelial lesions (HSILs) or worse. The cumulative incidence rates (CIR) were 29.7% (CI 24.8–30.1) for HSIL or worse among HPV mRNA-positive women at enrolment (39.5% 107/271) and 0% among HPV mRNA-negative women (60.5%, 164/271), (*p* = 0.002).

**Conclusions:**

Postmenopausal women with normal cytology testing positive for HR-HPV mRNA are at increased risk for the development of high-grade cervical intraepithelial neoplasia (CIN), in contrast to women with a negative HR-HPV mRNA outcome. The HR-HPV mRNA APTIMA assay detecting 14 HR-HPV types may be a useful triage method among HPV DNA-positive postmenopausal women with normal cytology.

## Background

International consensus on when to stop cervical cancer screening among postmenopausal women does not exist [1]. In Sweden, 30% of cervical cancer cases are diagnosed in women older than 60 years of age (The Board of Health and Welfare (2015) Cancer incidence in Sweden 2014).

In a recent audit in the region of Skåne (southern Sweden) it was observed that 24% (31/177) of squamous cervical cancer (SCC) or cervical adenocarcinoma cases between 2016 and 2017 were over 65 years of age (Personal communication Gunilla Thorn, Department of Clinical Pathology and Genetics, Lund Sweden).

Most of the affected older women have symptoms at the time of diagnosis due to an advanced cancer stage, and the mortality is as high as 70% [2]. This data indicate that there is a need to identify postmenopausal women who are at risk of developing cervical intraepithelial neoplasia (CIN) or cancer before they leave the screening programme.

Among postmenopausal women it has been shown that combined screening of high-risk (HR) human papillomavirus (HPV) DNA testing and cytology offers a higher sensitivity than screening with cytology alone [3, 4].

Therefore, between 2013 and 2016 the organised cervical cancer screening programme in the Skåne region performed a double test, consisting of both HR-HPV testing and cytology in postmenopausal women aged 60–65 years. It was considered as a last control before leaving the screening program. The double test consisted of a liquid-based cytology (LBC) specimen which was co-tested for HR-HPV DNA. In HR-HPV DNA-positive women with normal cytology the LBC specimen was further investigated for the presence of HR-HPV mRNA. Among such women with normal cytology we recently reported, in a prospective, one year follow-up study, that the specificity of the HPV mRNA-based APTIMA assay (60.2%) was superior to that of the Luminex HPV DNA assay (42.3%) regarding the detection of cervical pre-cancer lesions, while the sensitivities were similar [5].

However, the aim of the present study was to prospectively evaluate if the presence of HR-HPV mRNA at enrolment could predict the future development of cervical abnormalities among HPV- DNA-positive, postmenopausal women with normal cytology over a four-year follow-up period. Another aim was to evaluate to what extent HR-HPV mRNA testing can be used as a triage method in postmenopausal women.

## Methods

Between 2013 and 2016, women 60–65 years of age, with normal cytology, and living in the southern region of Sweden (Skåne) (*n* = 5925) were tested for the presence of HR-HPV.

When the Exit test was introduced in 2013 the laboratory had access only to the MGP-PCR Luminex HPV DNA assay and it was therefore used as the primary HPV assay throughout the study.

Cervical HPV DNA positivity was found in 286 (4.8%) individuals with a mean age of 61.9 years (SD +/− 1.7).

**Fig. 1:**
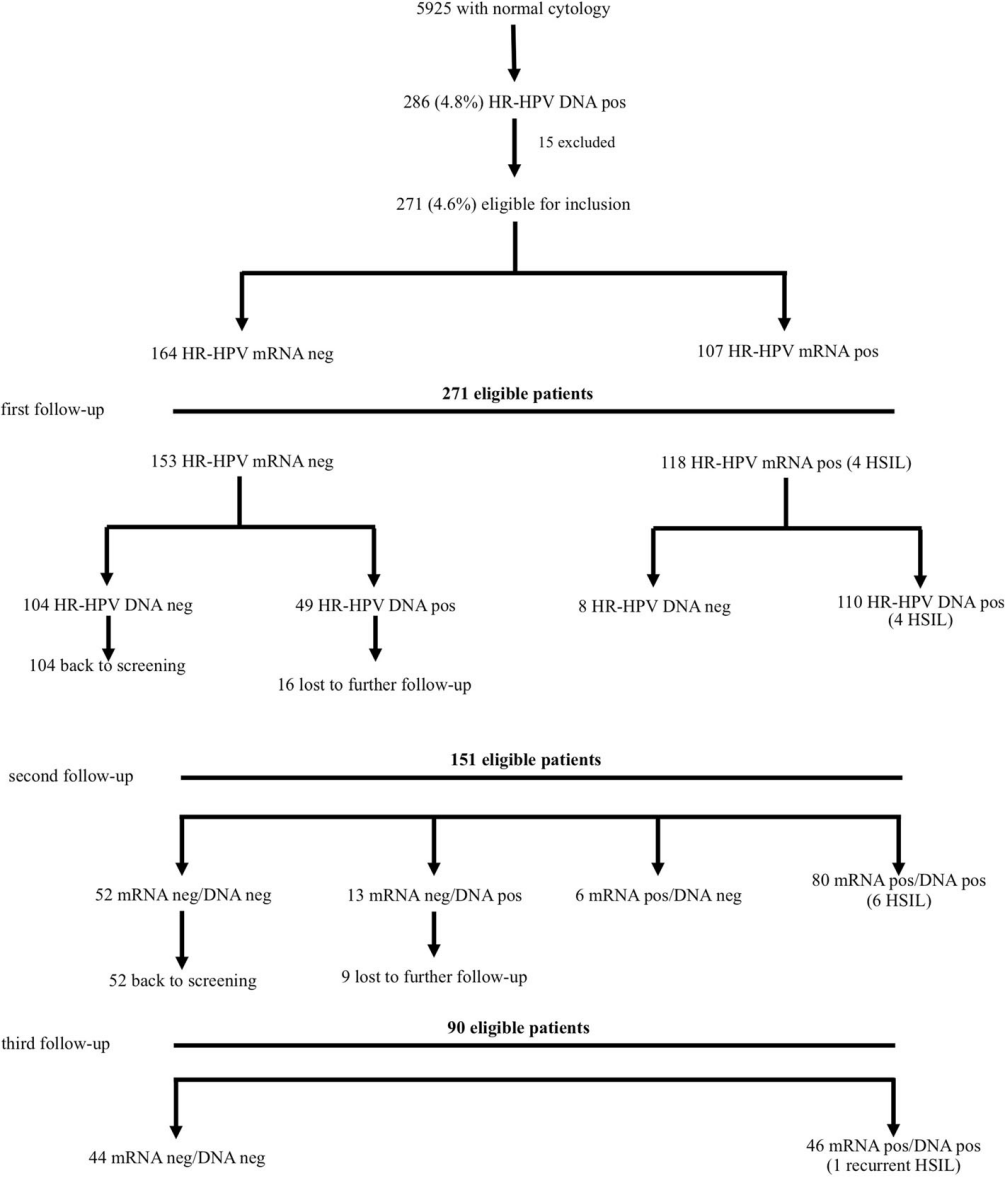
Inclusion process and follow-up data including HPV DNA and HPV mRNA outcomes

Criteria indicating exclusion from further follow-up were history of cervical neoplasia and/or treatment of cervical disease such as the loop electrical excision procedure (LEEP), hysterectomy or trachelectomy and ongoing oncological treatment at the time the double test was performed. A total of 271 HR-HPV DNA-positive women with normal cervical cytology were eligible for inclusion (Fig. 1) in this prospective follow-up study.

The double test consisted of an LBC sample (Thinprep, Hologic, Inc.) that was analysed for HR-HPV DNA by using the MGP-PCR-Luminex assay [6, 7]. In women testing positive for HR-HPV DNA, a concomitant HPV E6/E7 mRNA assay (APTIMA, Hologic, Inc.) was performed. Women with normal cytology and a positive HR-HPV DNA result were scheduled for a new follow-up examination after 12 months, including a new LBC specimen and a HPV DNA / mRNA co-testing procedure. All women diagnosed with cervical pathology and/or a positive HR-HPV DNA and/or mRNA result, were scheduled for a further clinical evaluation with colposcopic assessment. The same procedure was performed in women with a positive HR-HPV DNA and/or mRNA outcome on two subsequent controls. The next routine co-testing procedure was scheduled after 12 months, including even those women who had undergone a clinical examination.

At all further follow-up controls which were performed at intervals of 12 to 18 months, the same selection criteria were applied to determine which women were in need of a further clinical investigation. During our surveillance period at least three consecutive follow-ups could be documented.

Women presenting with normal cytology and negative HR-HPV DNA results left the routine screening service.

### Classification of LBC and histology results

LBC results were defined as normal, atypical squamous cells of undetermined significance (ASCUS), atypical glandular cells (AGCs), low grade squamous intraepithelial lesions (LSILs), and high-grade intraepithelial lesions (HSILs) according to the Bethesda classification [8]. Histopathological results were defined as LSIL and HSIL lesions using a two-tiered classification system [9]. Histologically confirmed HSIL or worse was used as the primary study endpoint. Low grade lesions based on cytological or histological findings are presented separately. Recurrent cytological abnormalities of the same severity level were considered as one incident case.

In women with HSIL lesions on LBC and corresponding colposcopic findings, an LEEP was performed for therapeutic management. Also, patients with cytological ASCUS or LSIL but a colposcopic picture suggestive of an underlying pre-cancerous lesion were scheduled for a LEEP procedure.

In the case of an inaccessible transformation zone located within the cervical channel, a cervical biopsy or conisation specimen was obtained for diagnostic reasons.

### HR-HPV testing

The MGP-PCR Luminex HPV DNA assay detects several HPV types simultaneously [6, 7].

From each LBC vial (Thinprep) 2 ml was centrifuged at 3500 *x g* for five minutes and then liquid was removed so that 500 uL remained. From each sample, DNA was purified by total NA-kit (200 uL input and 100 uL output) using Magna Pure LC (Roche) and then HPV DNA was amplified by PCR with modified GP5+/6+ (MGP) primers [7].

After amplification, the Luminex-based HPV genotyping allows the identification of the following HR-HPV types: 16, 18, 31, 33, 35, 39, 45, 51, 52, 56, 58, 59, and the probable high-risk type 68 (A and B) as well as the possibly high-risk types 26, 53, 66, 67, 69, 73 and 82 as described by IARC classification from year 2012 [10]. In the present study, probable and possible HR-HPV types were classified as HR-HPV types.

The HPV E6/E7 mRNA (APTIMA) assay detects qualitatively E6/E7 mRNA from 14 HR-HPV types: 16, 18, 31, 33, 35, 39, 45, 51, 52, 56, 58, 59, 66 and 68.

From each LBC vial (Thinprep) 1 ml was automatically transferred to 2.9 ml APTIMA transfer solution (ATS, Hologic, Inc.) by a Tomcat instrument (Hologic, Inc.). Thereafter an aliquot of 400 ul was further processed for the HPV E6/E7 mRNA (APTIMA) assay by the Panther system (Hologic, Inc.)

We calculated the proportion of HPV E6/E7 mRNA positivity for each of these 14 HR-HPV types as well as for HPV67 (APTIMA is known to cross-react with HPV67, Kit insert, APTIMA HPV Assay, nr 503,744), as determined by the MGP-PCR Luminex HPV DNA assay.

The presence of the same HR-HPV genotype at inclusion and at follow-up was defined as a persistent infection. At follow-up, for women with benign cytology who tested negative for HR-HPV DNA but were positive for low-risk (LR) HPV DNA according to the Luminex assay, the HR-HPV status was considered as negative or as cleared infection.

### Endpoints

The endpoint was the development of histologically confirmed HSIL or worse over a follow-up period of four years.

### Statistical evaluation

Statistical comparisons were based on two-sided chi-square tests. All comparisons were two-sided, and a 5% level of significance was applied. The absolute risk among among HPV mRNA positive women for the development of cervical abnormalities was presented with the corresponding 95% confidence intervals (CI).

Cumulative incident rates (CIR) during the follow- up period were calculated according to Kaplan-Meier survival analysis and presented as percentages with the corresponding 95% CI.

The statistical analyses were performed using SPSS version 19.0 or higher (IBM Corp., Amonk, NY, USA) and Omnistat (SBU, Trelleborg, Sweden).

## Results

Over a total surveillance period of 49 months (SD 11.0), the detection rate of histologically confirmed HSIL or worse was 3.6% (10/271).

Low grade lesions were detected in 13.3% (36/271) of the women. In 33 cases the diagnosis of ASCUS or LSIL lesions was based on cytological results and in the remaining three cases it was histologically confirmed.

**Fig. 2:**
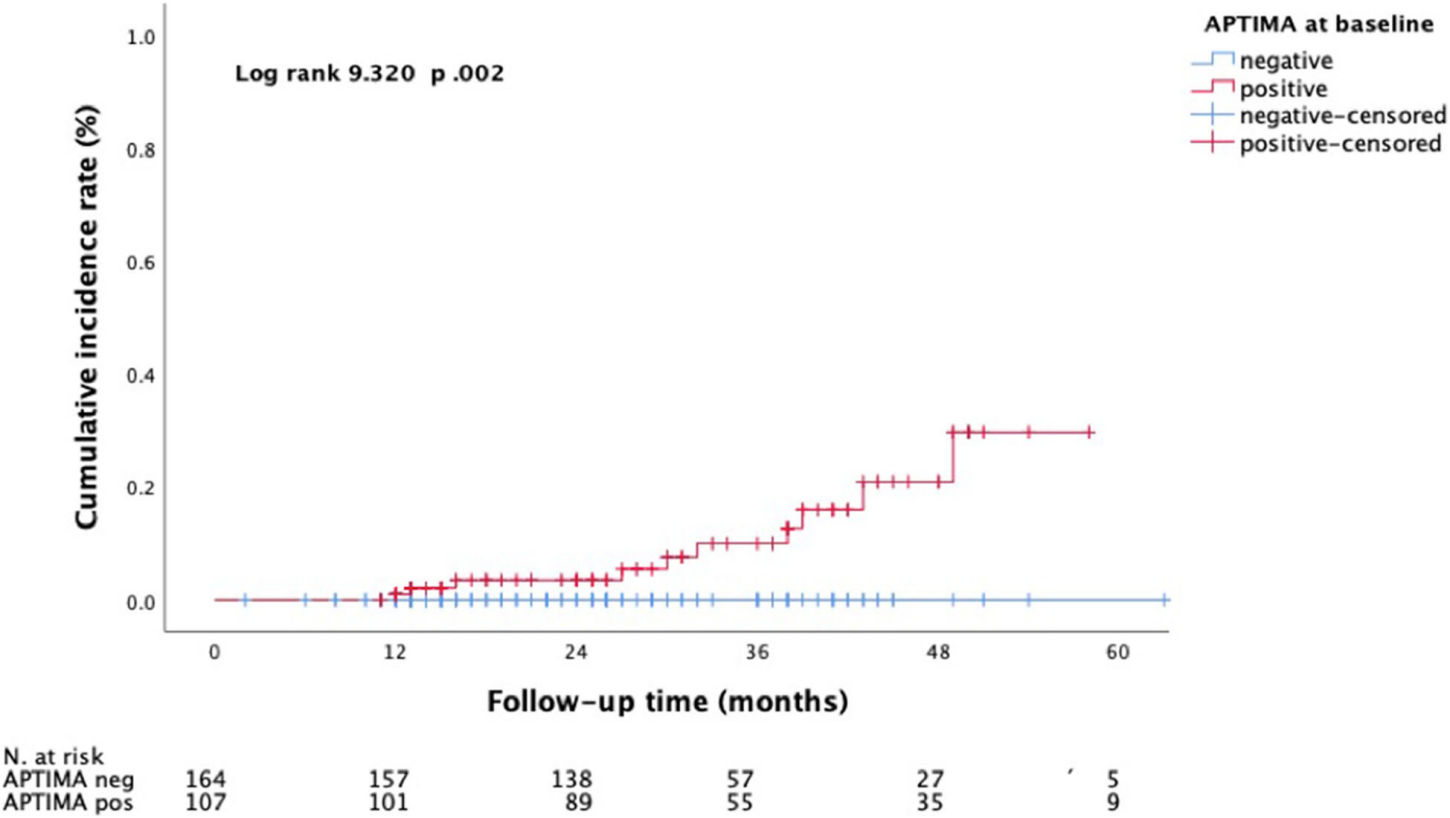
Incident cases of high-grade squamous intraepithelial lesions (HSIL) or worse over a mean follow-up period of 49 months (SD 11.0) in correlation to the HPV mRNA status obtained with the APTIMA assay at baseline

**Fig. 3:**
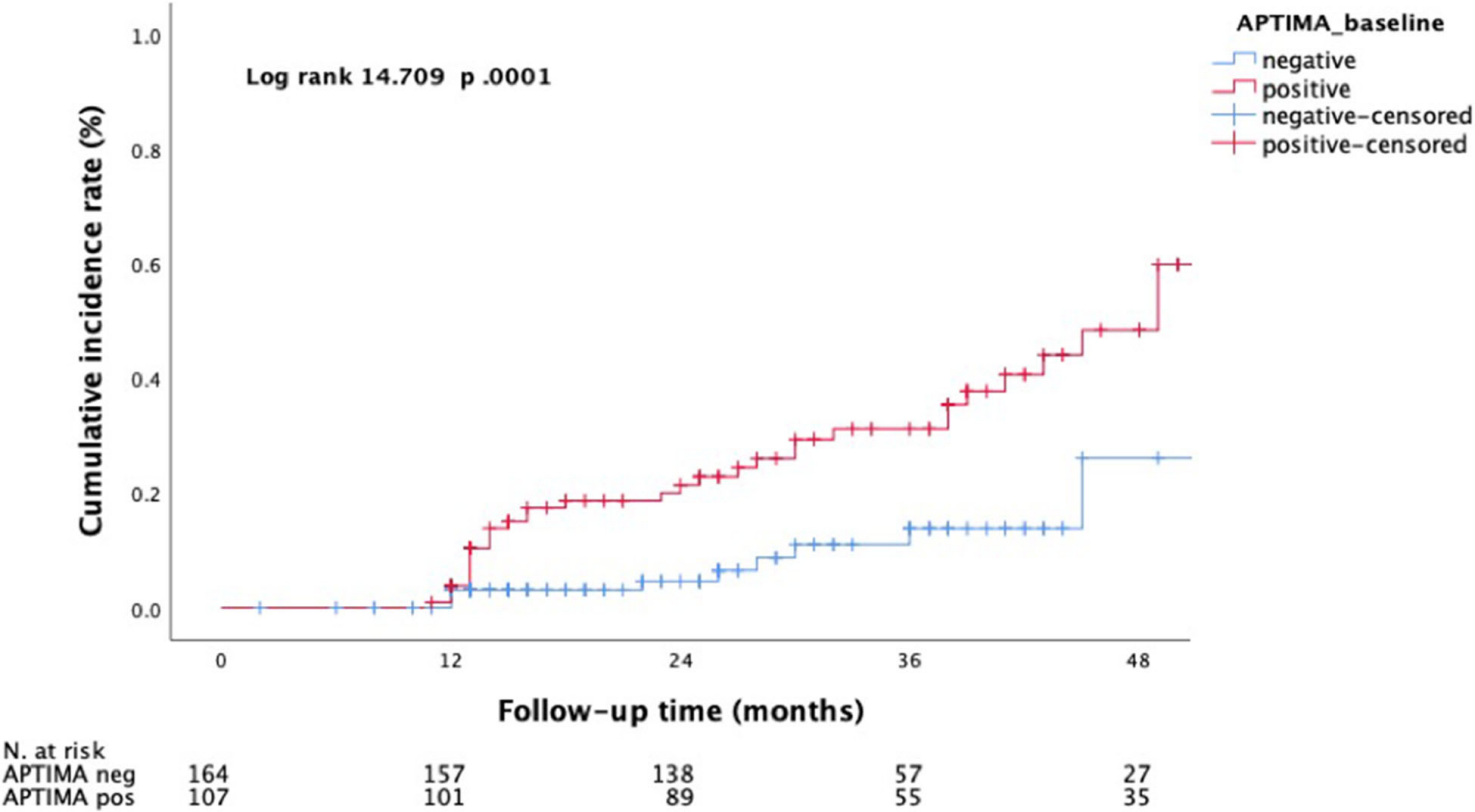
Incident cases of atypical squamous cells of undetermined significance (ASCUS) or worse over a mean follow-up period of 49 months (SD 11.0) in correlation to the HPV mRNA status obtained with the APTIMA assay at baseline

The CIR for HSIL or worse was 29.7% (95% CI 24.8–30.0) in the subgroup of HR-HPV mRNA-positive individuals and 0% in those women with a negative HR-HPV mRNA result (*p* = 0.002) (Fig. 2 & Table 1). The corresponding CIRs for ASCUS or worse were 59.9% (95% CI 57.3–66.2) and 26.1% (95% CI 16.5–35.9), respectively (*p* = 0.001) (Fig. 3).

**Table 1:** Cumulative incidence rate (95% CI) for HSIL and ASCUS based on HPV mRNA status and HR-HPV DNA types at baseline

CIR	HPV RNA pos	HPV mRNA neg	HPV DNA 16/18	HPV DNA other than 16/18
HSIL positive	29.7% (24.8–30)	–	14.6% (13.3–22.7)	18.5% (13.2–23.7)
ASCUS positive	59.9% (57.3–66.2)	26.1% (16.5–35.9)	64.9% (47.5–72.9)	51.1% (43.1–63.8)

*ASCUS* Atypical squamous cells of undetermined significance, *HSIL* high-grade squamous intraepithelial lesion, *HR* high risk

The absolute risk for the development of HSIL or worse was 9.3% (10/107, 95% CI 4.8–18.4) among HR-HPV mRNA positive women and 0% (0/164, 95% CI 0–27.5) among those individual with a negative HR-HPV mRNA result at baseline. The corresponding figures for ASCUS or worse were 31.8% (34/107, 95% CI 23.7–41.1) and 7.31% (12/164, 95% CI 4.12–12.5) respectively.

At baseline, the overall HPV mRNA positivity rate was 39.5% (107/271) and HR-HPV mRNA was present in all cases of histologically confirmed HSIL and in 67% (24/36) of the women diagnosed with ASCUS or LSIL.

Among women testing positive for HR- HPV DNA types 16/18 the CIR for HSIL or worse was 14.6% (95% CI 13.3–22.7) and among those with other HR-HPV DNA types the corresponding figure was 18.5% (95% CI 13.2–23.7, *p* = 0.68). The CIRs for ASCUS or worse were 64.9% (95% CI 47.05–72.9) for women infected with HR-HPV DNA types 16/18 and 51.1% (95% CI 43.05–63.8, *p* = 0.67) in the other subgroup (Table 1).

After one year, 41.3% (112/271) of the HR-HPV DNA-positive women had cleared their infection, whereas 59% (159/271) were still persistently infected with the same HR-HPV DNA type(s). Over the entire follow-up period, persistent HR-HPV type(s) were observed among 16.9% (46/271) of the women whereas clearance of the HR-HPV infection was found in 73.8% (200/271) of the cases. Twenty-five women were lost during follow-up after two years (see Fig. 1).

HPV16, 68A, 31, 52 were the most common HR-HPV types at enrolment (Table 2). At inclusion, 71% (193/271) had a single type infection according to the DNA-based Luminex assay while 29% (78/271) had double or triple infections with other HR- and/or LR HPV types (data not shown). Among the 46 women with a persistent HR-HPV DNA infection at long-term follow-up, 21 (44%) were found to have infections, with a least two HR-HPV DNA types at baseline.

**Table 2:** Frequencies of Human papillomavirus genotypes in women with HPV DNA-positive and normal cytology at baseline in relation to the worst findings on cytology/histology at follow-up

Cytology/Histology at follow-up (n)				
HPV types baseline	Normal findings (*n* = 19 histologically confirmed)	LSIL/ASCUS (*n* = 3 histologically confirmed)	HSIL (*n* = 10 histologically confirmed)	Total
16	42	5	2	49
68A	22	3	1	26
31	19	3	2	24
52	19	4	0	23
51	16	1	1	18
66	15	7	0	22
56	13	3	1	17
39	13	1	1	15
18	13	2	1	16
45	11	2	0	13
58	6	1	0	7
35	5	0	0	5
59	4	0	0	4
68B	2	0	0	2
33	1	0	0	1
Other	24	4	1^a^	29
Total	225	36	10	271

*ASCUS* Atypical squamous cells of undetermined significance, *HSIL* high-grade squamous intraepithelial lesion, *LSIL* low grade intraepithelial lesion

^a^ Among the group of other HR-HPV types, HR-HPV 67 was detected in the case diagnosed with an HSIL lesion

Among 153 women with a negative HR-HPV mRNA result at the first follow-up control, 77% (104/153, 95% CI 84.9–126.0) had no longer detectable HPV DNA, whereas the corresponding clearance rate among the HPV mRNA-positive women was 7% (8/118; 95% CI 3.4–15.6, OR: 29.2, *p* < 0.0001) (see Fig. 1 & Table 3).

**Table 3:** Clearance and persistency rates of HR-HPV infections at the different follow-up intervals with the corresponding Odds ratio and *p*-values

Follow-up interval (Number of evaluated patients and their types of HR-HPV infection)	Clearance rate (%)^a^	Persistency rate (%)^**b**^	Odds ratio (95% CI)	***P***-value
I follow-up (Total Number *n* = 271)				
HPV mRNA pos/ DNA pos = 110	*n* = 112 (41.3%)	*n* = 159 (58.6%)	29.2 (13.2–64.6)	< 0.0001
HPV mRNA pos /DNA neg = 8				
HPV mRNA neg/DNA pos = 49				
HPV mRNA neg/DNA neg = 104				
II follow-up (Total Number *n* = 151)				
HPV mRNA pos/ DNA pos = 80	*n* = 58 (38.4%)	*n* = 93 (61.6%)	3.23 (1.4–7.2)	0.004
HPV mRNA pos /DNA neg = 6				
HPV mRNA neg/DNA pos = 13				
HPV mRNA neg/DNA neg = 52				
III follow-up (Total Number *n* = 90)				
HPV mRNA pos/ DNA pos = 46	*n* = 44 (48.4%)	*N* = 46 (51.2%)	10.3 (0.5–197.8)	0.12
HPV mRNA neg/DNA neg = 44				

^a^Including HR-HPV mRNA and/or HPV DNA-negative patients

^b^ Including HR-HPV mRNA and/or HPV DNA-positive patients

At the second follow-up control, 20 out of the 33 women (60.6%; 95% CI 12.1–30.8) who remained HR-HPV mRNA-negative but HPV DNA-positive after the first control, were found to have negative co-testing results. The corresponding figure among the HR-HPV mRNA-positive women was 32% (38/118, 95% CI 26.9–52.1, OR 3.2, *p* = 0.004) (see Fig. 1 & Table 3).

The LEEP procedure was performed in all 10 patients with cytological HSIL and in seven patients with ASCUS or LSIL, due to abnormal findings on colposcopy. Histopathological evaluation revealed seven cases of HSIL, two glandular pre-cancer lesions and one case of cervical adenocarcinoma. In other 22 patients with cytological ASCUS or LSIL, a diagnostic conisation procedure was able to confirm the presence of histopathological LSIL in three cases and benign tissue conditions in the remaining 19 patients. In seven patients with cytological low grade lesions, no tissue material was obtained due to normal findings on colposcopy.

## Discussion

In our cohort of HR-HPV DNA-positive postmenopausal women with normal cytology we were able to detect 10 cases of histologically confirmed HSIL lesions. Another 36 women were diagnosed with ASCUS or LSILS lesions based on either cytological (*n* = 33) or histological (n = 3) results.

Women who tested positive for HR-HPV mRNA at enrolment were found to have a significantly increased risk for HSIL or worse (CIR 29.7%) and also for ASCUS or worse (CIR 59.9%) over a total surveillance period of 49 months.

In contrast, none of the HPV DNA-positive women with a negative HR-HPV mRNA result at baseline developed HSIL or worse (CIR 0%) during follow-up, although a risk regarding the development of low grade lesions such as ASCUS or LSIL was still evident (CIR 26.1%). Concerning the persistency of HR-HPV types we observed that after four years of follow-up, 16.9% of the women were still diagnosed with a persistent HR-HPV infection while 73.8% had cleared their infection spontaneously.

It is already shown in the literature that HR-HPV testing is a safe screening option in postmenopausal women [11] as it increases the likelihood of identifying cervical pre-cancer lesions while cytology alone is known to have a relatively low sensitivity in this age group [3] [12].,

Also according to the current Swedish screening guidelines, women 30 years or older are screened with HR-HPV testing until the age of 64 while cytology is the screening method of choice in women 23 to 29 years old. A double test including cytology and HR-HPV testing is only indicated in women at 41 years of age. Only women with at least one negative HR-HPV DNA test at the age of 64 are allowed to exit the screening programme.

Even though most of the HR-HPV assays approved for primary cancer screening are based on HR-HPV DNA detection, like the Hybrid Capture 2 (HC2) method or the GP5+/6 + EIA assay or the COBAS® there is now growing evidence that HR-HPV detection methods targeting the mRNA of the oncoproteins E6/E7 are also effective and reliable alternative screening tools [13–18].

A further indicator underlining the limited sensitivity of cytology in the postmenopausal age group is the observation that we were able to detect a discrepancy of about 20% between LBC results and the corresponding histological findings in our series. In three women with ASCUS or LSIL, histopathological analysis of the matching LEEP specimen revealed the presence of underlying HSIL lesions, while in three other women with high-grade cytology no pre-cancerous findings could be confirmed on conisation material. According to the literature, the discrepancy level between cytology findings and the corresponding histological outcomes varies between 5 to 55%. Factors that may cause those elevated rates of false negative results are the subjective interpretation of the specimen and/or the absence of diagnostic cells [19, 20].

A clinical circumstance that may further contribute to the limited sensitivity of cervical cytology is the higher probability of sampling errors as the transformation zone tends to be located higher up in the cervical channel. Furthermore, ageing effects such as a decline in oestrogen can lead to the presence of atrophic cells which in their turn can be mistakenly interpreted as ASCUS or LSIL lesions [20, 21]. The described difficulties in obtaining an adequate LBC sample in postmenopausal women underline the need for an objective screening tool in this age group offering a higher sensitivity, i.e. HPV analyses.

Our data show that HR-HPV mRNA-positive women aged 60 years or older are at risk of developing cervical abnormalities and are therefore in need of regular follow-up controls including HPV mRNA analyses. Additional gynaecological examination is indicated if a persistent HR-HPV mRNA infection is found at two subsequent annual controls and/or cytology shows abnormal results. Also, Johannson et al. demonstrated that among HPV DNA-positive women aged 35 years or older with either cytological ASCUS or LSIL at baseline, a positive HR-HPV mRNA result could predict the development of CIN 3 or worse with a sensitivity of 100% within the following four years [22]. As in our study, all women who were diagnosed with CIN 3 or worse were HPV mRNA-positive at baseline.

Postmenopausal women represent a special patient group, as they tend to have a higher risk for persistent HR-HPV infections than younger individuals, who have a higher acquisition frequency but also a faster clearance rate [23, 24]. Furthermore a type-specific HR-HPV persistence, especially for HR-HPV types 16, 18 and 31, appears to be associated with the future development of cervical pre-cancer or worse in this age group [11].

It is of clinical importance to distinguish between those individuals with active viral replication who are at risk of developing cervical pre-cancer lesions and those with latent HR-HPV DNA infections lacking any clinical significance.

There is evidence in the literature that the level of the HPV mRNA copies increases proportionally to the severity of the cervical lesion [25]. On the other hand, a negative HPV mRNA result in combination with a positive HPV DNA outcome seems to reflect the presence of an inactive HR-HPV infection with low or absent viral replication. According to our data, a negative HR-HPV mRNA result at the beginning of the follow-up process was associated with a high probability of clearing an existing HPV infection spontaneously within a year. In our series, a total of 104 out of 153 women (77%) testing negative for HR-HPV mRNA but positive for HPV DNA at the beginning of the follow-up process showed negative co-testing results at the first control (see Fig. 1). Among the women who initially were found to be HR-HPV mRNA-positive, only eight individuals had healed out their HR-HPV DNA infection. Therefore, the total clearance rate within the first 12 months was 41.3% (112/271).

Those data are comparable to the results of other studies reporting clearance rates of about 40% in older women within an average time span of four months [4, 11, 26]. The data of recent studies indicate that the long-term protective effect of a negative HPV mRNA result is comparable to that of a negative HPV DNA test [18, 22, 27].

**Fig. 4:**
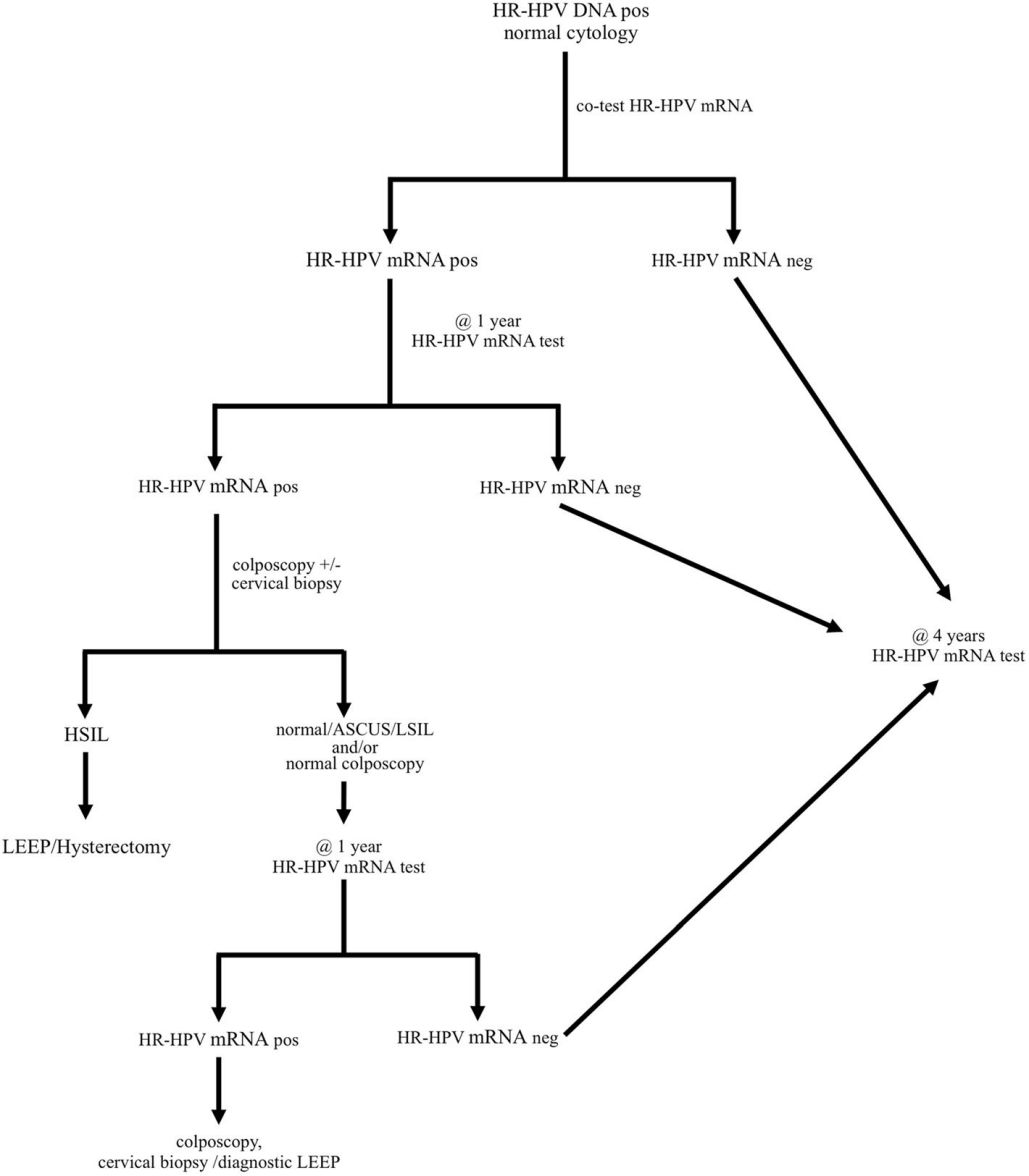
Suggested algorithm for using HR-HPV mRNA testing as a triage method in women exiting the screening program

A cohort study with long-term follow-up was able to demonstrate that the five-year cumulative risk of developing CIN 3 or worse was comparable between the cohort of HPV DNA-negative and HPV mRNA-negative women [28]. Those clinical data lead to the assumption that the implementation of four-year screening intervals is a safe strategy among HR-HPV mRNA-negative women (Fig. 4), similar to HR-HPV DNA-negative women aged 40 years or older [18, 29, 30].

Regarding the risk stratification of cytological ASCUS and LSIL lesions, it is stated in the literature that the APTIMA assay offers a higher specificity when compared to other DNA based HR-HPV detection methods [13, 16, 31]. This is in agreement with the observation that in our series none of the 12 ASCUS lesions detected in HPV mRNA-negative women was of clinical significance. In all cases the LBC results at follow-up were classified as benign and cervical biopsy was able to confirm the presence of normal underlying tissue conditions.

Furthermore, the results of a long-term follow-up study demonstrated that a negative APTIMA co-testing result among HPV DNA-positive women with minor cytological abnormalities at baseline was associated with a high negative predictive value of 100% for CIN 3 or worse within the following four years [22]. Even though it seems that HPV mRNA negative women with cytological ASCUS or LSIL might be re-screened safely at four-year intervals, further long-term studies are necessary to determine if these assumptions can also be applied to the postmenopausal patient cohort.

Regarding the distribution of the HR-HPV genotypes, we observed that HR-HPV types 16, 68A and 31 were the most frequent ones in our cohort. We observed a relatively high frequency of HPV68A infections (9.5%), which is rather unusual in younger women and not covered by the current vaccination programmes. Those data are in line with other studies showing that the HR-HPV profile in postmenopausal women to some extent differs from those encountered in younger individuals [32, 33]. Furthermore, a study of the HR-HPV profile in postmenopausal women demonstrated that HPV31 contributed more to the development of cervical dysplasia than HPV16/18 [34].

## Conclusions

Our data favour the use of HPV mRNA detection as triage method in HPV DNA-positive postmenopausal women with normal cytology (Fig. 4).

Since postmenopausal women testing positive for HR-HPV mRNA and normal cytology have a substantial risk (9.3%) of developing severe cervical abnormalities we suggest that these women should be scheduled for regular, annual follow-up examinations. In the case of a persisting HR-HPV mRNA infection at two subsequent controls and/or the presence of cytological abnormalities, a cervical tissue biopsy/curettage or diagnostic LEEP specimen should be obtained, especially if the transformation zone is inaccessible for colposcopic evaluation.

On the other hand our data indicate that postmenopausal women with a negative outcome for HR-HPV mRNA are no longer in need of further follow-up for at least four years.

## Data Availability

The data of the cervical cancer screening registry are stored at the regional cancer centrum of the Skåne region (RCC Syd; www.cancercentrum.se) including also the HPV results obtained at the department of microbiology at the Lund university. For information about the data and/or access to the raw-database contact the corresponding author Katrin Christine Asciutto (christine.asciutto@yahoo.com; postal address: Wedemhove 74, 48157 Muenster, Germany).

## Abbreviations

ASCUS: Atypical squamous cells of undetermined significance
CIN: Cervical intraepithelial neoplasia
CIR: Cumulative incidence rate
HR-HPV: High-risk human papilloma virus
HSIL: High-grade squamous intraepithelial lesion
LBC: Liquid-based cytology
LEEP: Loop electrical excision procedure
LSIL: Low grade squamous intraepithelial lesion
